# New Views on Respiration

**Published:** 1852-06

**Authors:** 


*New Views on Respiration.*—Prof. Draper proposes, in the April number of the *American Journal of the Medical Sciences*, some modifications of the received theory of respiration. He contends, first, for the necessity of the constant action of the circular organic muscles; second, for the condensing action of the three tissues—the wall of the pulmonary vesicle, the pulmonary capillary, and of the blood-disk; and third, conjectures that there is an analogy between the relation to oxygen of hematine, and another nitrogenized coloring principle, indigo. His views are thus summed up:

The air introduced by atmospheric pressure, brought into play by the action of the diaphragm and other respiratory muscles, fills, in ordinary respiration, the nasal passages, trachea, and larger ramifications of the bronchial tubes. Between it and the gas coming from the pulmonary vesicles, diffusion steadily takes place, tending to remove the cell-gas into the atmosphere; but this gas is not brought from the vesicles by diffusion, which could not act with sufficient speed, but by the contraction of the circular organic muscles of the bronchial tubelets and of the cells, the different bronchial trees not acting simultaneously, but successively. As soon as the contraction is over, the tubes expand by their elasticity, and air is drawn into the cells. It is probable that, in producing these results, the vibratile cilia conspire, and the effect is aided by contemporaneous contraction of other bronchial trees, and the whole process ends with the expulsion of the foul air which has accumulated in the larger bronchi and trachea by the diminution which ensues in the general capacity of the chest during expiration. In respiration, the lungs are not, therefore, passive, as is commonly said.

The exchange between the gas in the cells and that in the blood does not occur through simple diffusion, or in quantities proportional to the diffusion volumes of the oxygen and carbonic acid. It is a complex diffusion, in which the disturbances arise from the gases in the blood being either dissolved or combined; and through three intervening membranes, that of the air-cells, of the pulmonary capillary, and of the blood-disk, all of which exert a condensing action, of the result of which it is impossible to furnish any numerical estimate.

Brought into presence of the hæmatin, the oxygen may possibly associate itself therewith, in a manner analogous to that which we witness, under similar circumstances, with deoxidized indigo.

